# White blood cell counts and neutrophil to lymphocyte ratio in the diagnosis of testicular cancer: a simple secondary serum tumor marker

**DOI:** 10.1590/S1677-5538.IBJU.2014.0593

**Published:** 2016

**Authors:** Ozgur Haki Yuksel, Ayhan Verit, Aytac Sahin, Ahmet Urkmez, Fatih Uruc

**Affiliations:** 1 Department of Urology, Fatih Sultan Mehmet Research & Training Hospital, Istanbul, Turkey

**Keywords:** NLR protein, mouse [Supplementary Concept], Testicular Neoplasms, Biomarkers, Tumor

## Abstract

**Purpose:**

The aim of the study was to investigate white blood cell counts and neutrophil to lymphocyte ratio (NLR) as markers of systemic inflammation in the diagnosis of localized testicular cancer as a malignancy with initially low volume.

**Materials and Methods:**

Thirty-six patients with localized testicular cancer with a mean age of 34.22±14.89 years and 36 healthy controls with a mean age of 26.67±2.89 years were enrolled in the study. White blood cell counts and NLR were calculated from complete blood cell counts.

**Results:**

White blood cell counts and NLR were statistically significantly higher in patients with testicular cancer compared with the control group (p<0.0001 for all).

**Conclusions:**

Both white blood cell counts and NLR can be used as a simple test in the diagnosis of testicular cancer besides the well-known accurate serum tumor markers as AFP (alpha fetoprotein), hCG (human chorionic gonadotropin) and LDH (lactate dehydrogenase).

## INTRODUCTION

All of the cells involved in the immune response are formed via differentiation from pluripotent hematopoietic stem cells of the bone marrow. From common lymphoid progenitor cells among early stage precursor cells, T and B cells of the adaptive immune system and natural killer (NK) cells of natural immunity differentiate. Common myeloid progenitor cell which is another early stage precursor cell firstly differentiates into granulocyte/macrophage cells, then through a series of differentiation phases, they induce formation of dendritic cell, granulocytes (neutrophils, eosinophils, basophils and mast cells), monocyte macrophage cells involved in natural immunity. Immune response triggered against various components of microorganisms, macromolecules as protein and polysaccharides or even small chemical components can protect the organism or even lead to deleterious outcomes. As a criteria for the degree of natural and adaptive immune response against antigenic formations as cancer cells, neutrophil to lymphocyte ratio (NLR) can be used.

Testicular cancer (Ca) is a relatively rare disease, accounting for one percent of all neoplasms in men. Despite its low incidence, the investigation of testicular cancer is important because this malignancy occurs in a relatively young male population of 15-35 years. The fact that testicular cancer is the most curable adult solid tumor, irrespective of the tumor spreading, opens the question of immunological influence on such a favorable outcome. Reports on immunocompetence in testicular cancer patients are scarce.

The role of NLR in the evaluation of progression-free survival and pre-and postoperative treatment of various oncological cases including urogenital and non-urogenital tumors have been investigated in many studies. In our study, preoperative NLR’s and neutrophil counts of patients with localized testicular tumors and that of varicocele patients who were included in the study as a control group, were compared. In this pioneer study, we investigate the role of NLR and neutrophil counts in localized testicular Ca in patients with low tumor volume.

## MATERIALS AND METHODS

This retrograde study was performed on 72 male (36 testicular cancer, and as a control group 36 varicocele patients) patients. Informed consent forms were taken and institutional review board was approved from hospital ethics committee. Age, number and percentage of neutrophils and lymphocytes, hemoglobin levels and NLR’s (neutrophil count divided by the number of lymphocytes; neutrophil-lymphocyte ratio was calculated) in peripheral blood samples obtained during preoperative period were analyzed in detail and subjected to statistical analysis. Patients with an evidence of concomitant infection or inflammation were excluded from the study.

### Statistical analysis

For statistical analysis, NCSS (Number Cruncher Statistical System) 2007&PASS (Power Analysis and Sample Size) 2008 Statistical Software (Utah, USA) program was used. Study data were evaluated using descriptive statistical methods (mean, standard deviation, minimum, maximum, median, frequency, and ratio). In the intergroup comparisons of quantitative data, for parameters demonstrating normal distribution Student t test and, for those without normal distribution Mann Whitney U test were used. Statistical significance was rated at p<0.01 and p<0.05 respectively. The areas under the receiver operating characteristic curves (ROC) were used to assess the discriminative ability of NLR and neutrophil counts in localized testicular Ca.

## RESULTS

**Figure 1:**
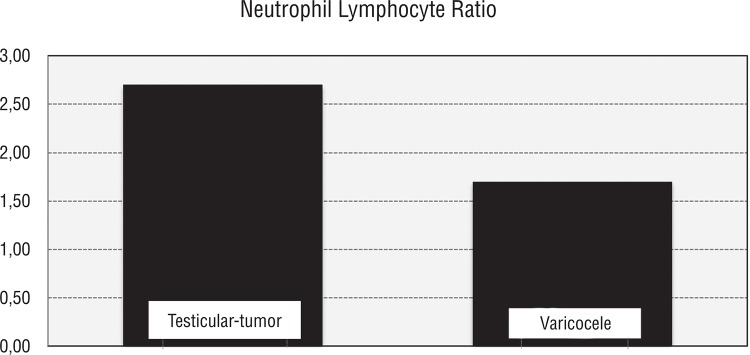
Distribution of neutrophil/lymphocyte ratios between groups.

**Figure 2:**
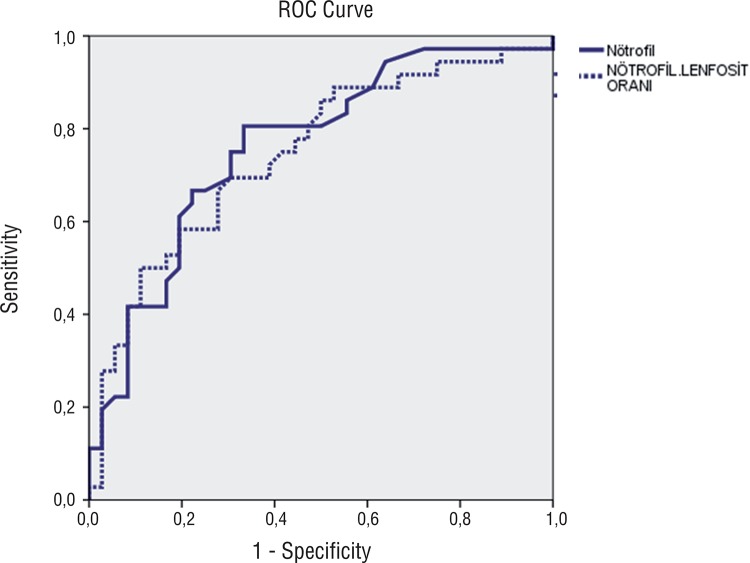
ROC of NLR and neutrophil counts in localized testicular cancer.

Thirty-six patients with localized testicular cancer with a mean age of 34.22±14.89 years and 36 healthy controls with a mean age of 26.67±2.89 years were enrolled in the study. The pathological subtypes of the study group consisted of seminomas (n: 6), mixt germ cell carcinoma (n: 18), embryonic carcinoma (n: 7), teratomas (n: 4) and Leydig cell carcinoma (n: 1). Localized testicular Ca is defined as; up to pT2 N0 M0. Ages and blood values and their distribution with respect to groups are shown in Tables 1 and 2, respectively. Statistically and extremely significant intergroup differences were found as for neutrophil counts and percentages (p=0.001 and p<0.01 respectively). Neutrophil counts and percentages noted in patients with testicular tumors were significantly higher when compared with varicocele patients. Neutrophil percentages were statistically and extremely different between both groups (p=0.001 and p<0.01 respectively). Neutrophil percentages of patients with testicular tumors were statistically significantly higher than those of the patients with varicoceles. Lymphocytic measurements did not show a statistically significant difference between groups (p>0.05). A statistically and extremely significant intergroup difference was detected between percentages of lymphocytes (p=0.001 and p<0.01 respectively). Percentages of lymphocytes in patients with testicular tumors were significantly lower. A statistically and extremely significant difference was found between groups as for NLR’s of the cases (p=0.001 and p<0.01 respectively). NLR’s in cases with testicular tumors were significantly higher than in patients with varicocele (Figure-1). A statistically and extremely significant difference was detected between groups with respect to estimated NLR (p=0.001 and p<0.01 respectively). NLR’s in patients with testicular tumors were significantly higher relative to those seen in patients with varicoceles. A statistically significant difference between groups as for hemoglobin levels was not detected; hemoglobin measurements in patients with testicular tumors were notably lower than those of varicocele patients (p=0.099 and p>0.05 respectively). The area under ROC curve for NLR in localized testicular cancer patients was 0.74, (95% confidence interval (CI)=0.63-0.86), with a threshold value of 2.06 and sensitivity =69 % and specificity=69%. The area under ROC curve for neutrophil counts in localized testicular cancer patients was 0.76, (95% confidence interval (CI)=0.65-0.87), with a threshold value 4.40(K/µL) and sensitivity=80% and specificity=66% (Figure-2).

**Table 1:** Distribution of age and hematological values.

		Min-Max	Mean±SD
Age (years)		17.0-48.0	30,44±11,31
Neutrophil counts (K/µL)		2.6-11.0	4.99±1.81
Neutrophil ratio (%)		38.9-84.6	60.71±10.55
Lymphocyte counts (K/µL)		0.9-3.7	2.22±0.68
Lymphocyte ratio (%)		10.3-47.8	28.59±9.07
Neutrophil/lymphocyte ratio (%)		0.87-8.50	2.58±1.60
Neutrophil/lymphocyte ratio (%)		0.86-8.21	2.56±1.57
Body mass Index (kg/m^2^) (n=36)		20.3-35.0	25.71±3.39
Hemoglobin (gr/dL)		12.1-17.5	14.89±1.09
B-HCG (mIU/mL) (n=32)		0.5-1987.0	137.17±459.89
AFP (U/mL) (n=32)		1.2-1656.0	111.70±342.18
LDH (U/L) (n=32)		106.0-954.0	194.19±146.49
		**n**	**%**
Group	Testicular tumor	36	50
	Varicocele	36	50

**Table 2:** Intergroup comparisons of patients’ ages and hematological values.

	Testicular tumor (n=36)	Varicocele (n=36)	^a^p
	Mean±SD	Mean±SD	
Neutrophil counts (K/µL)	5.78±1.93	4.21±1.27	0.001**
Neutrophil ratio (%)	64.84±11.04	56.60±8.31	0.001**
Lymphocyte counts (K/µL)	2.11±0.74	2.33±0.59	0.16
Lymphocyte ratio (%)	25.06±8.77	32.11±8.03	0.001**
Hemoglobin (gr/dL)	14.68±1.08	15.10±1.07	0.099
Neutrophil/lymphocyte ratio; (Median)	3.18±1.76 (2.7)	1.99±1.17 (1.8)	^b^0.001**
Neutrophil/lymphocyte ratio (%) (Median)	3.12±1.72 (2.6)	2.01±1.18(1.7)	^b^0.001**

^a^Student-t Test

^b^Mann-Whitney U Test

**p<0.01

## DISCUSSION

Systemic inflammation can be measured by a variety of biochemical and hematological markers. Total leukocyte, neutrophil and lymphocyte count have been used as a marker of inflammation for many years. Neutrophils mediate inflammation by various biochemical mechanisms such as release of arachidonic acid metabolites and platelet aggravating factors (1). Neutrophilia could represent a consequence of ectopic production of myeloid growth factors as part of a paraneoplastic syndrome (2) or, more likely, a nonspeciﬁc response to cancer-related inﬂammation secondary to tissue destruction and cytokine releases. Experimental data indicate that activated neutrophils may directly and indirectly stimulate tumor growth (3). Lymphopenia is associated with cortisol induced stress response (1). NLR and platelet to lymphocyte ratio (PLR) have also been shown to be reliable markers of systemic inflammation that were provided by many studies (4). Cells with anti-tumoral activities belong to a wide spectrum including neutrophils, type-2-macrophages, plasmacytoid dendritic cells, suppressive cells derived from myeloid tissue, and mediator T cells (5).

Inﬂammation plays a critical role in many aspects of cancer, including tumor development, progression, clinical presentations and prognosis (6). The systemic inﬂammatory and immune responses to tumor cells and tumor cell-secreted peptides vary with the type and extent of malignancy. The tumor/host interaction may have signiﬁcant inﬂuence on patients’ outcome. However, this effect is generally not taken into account in current prognostic systems. There is now accumulating evidence that the markers of the systemic inﬂammatory response, including cytokines, C-reactive protein (CRP), albumin, serum amyloid A and white cell count, can be independent prognostic factors in cancer patients (6). Immune system has a dual function both in the development and progression of cancer. It can destroy tumor cells and on the other hand it can promote growth of active malignant cells, their invasive capacities and metastatic abilities. Excess number of neutrophils in the circulation has been conceived to have an important role in the tumor progression and angiogenesis. Therefore increased neutrophil counts should be related to poor prognosis (7).

Presence of cancer-related systemic inflammatory response has been evaluated in various oncological diseases including renal cell carcinoma, upper urinary tract cancers, bladder cancer, prostate cancer and many studies have demonstrated its effective role in the prediction of surgical margin positivity during postoperative period, and also progression-free survival.

Limited numbers of reports are available on immune resistance in patients with testicular cancer. Considerable evidence supports the view that the biological behavior of tumors and in particular, their capacity to metastasize are in part determined by immunological factors requiring participation of T lymphocytes, B lymphocytes, macrophages and natural killer cells. Immunological reactivity has been analyzed in a wide spectrum of solid tumors and a vast literature indicates a correlation between depressed cell-mediated immunity and the stage of the disease. On the contrary, there is little evidence about the role of immunological factors in the development and spread of testicular tumors.

In one of the recent studies at this topic, age, female gender, NLR and platelet counts were found to be invasive determinants of urothelial carcinoma. In this study, threshold value for NLR was accepted as 2.5 (8). In our study, NLR in the group with testicular cancer was estimated as 3.18±1.76 (20.7).

In another study, NLR has been indicated as a potentially important criterion in urothelial carcinoma for the detection of extravesical disease (9). Also, NLR has been suggested as one of the factors indicating poor prognosis.

In a study which established cut-off value of NLR as 2.7, the authors indicated that combination of T stage, and NLR could be used for the stratification of recurrence risk in patients with non-metastatic renal cell carcinomas (10). Higher NLR before treatment has been related to poor prognosis for various types of cancer including renal cell carcinoma. In a study on metastatic renal cell carcinoma, it has been reported that decreased NLR might be a criterion for progression-free survival, and treatment response and also an indicator of the balance between host immunity and cancer-related inflammation (11).

Similar studies have been also performed in non-metastatic upper urinary system cancers. In these studies performed using directly neutrophil counts, the value of relevant data for patient counseling and identification of patients with poor prognosis for neoadjuvant chemotherapy has been indicated (12).

Unlike the present one, in previous studies the study groups involved the same oncologic diagnosis within different stages to evaluate the treatment success and as a prognostic factor, however our study design structured in mostly composed of low stage testis cancer patients and had a control group. Thus we claimed that NLR can be added as a helper diagnostic marker of testis cancer. In the unique study with a similar design that analyzed C-reactive protein levels in testicular cancer, the authors demonstrated that C-reactive protein in testicular cancer can be an important marker in the prediction of the development of secondary non-germ cell cancer which has been accepted as a late-term complication of testicular tumor (13).

An association between high NLR and increased mortality or recurrence has been observed in various solid organ tumors, including lung, pancreatic, hepatocellular and cholangiocarcinoma (14-17).

Testicular cancer is an endocrine malignancy like thyroid cancer. It was reported that higher NLR was associated with increased tumor size and high ATA risk of recurrence in patients with differentiated thyroid cancer (18).

Neutrophils are the first line of natural immune defense against inflammation. Neutrophils may lead to increased endothelial permeability by releasing vasoactive and cytotoxic agents such as reactive oxygen species and digestive proteases during inflammation. Neutrophils contribute to IL-2 induced vascular leak syndrome. It has been also reported that vascular endothelial growth factor (VEGF) has positive correlations with neutrophil and NLR and negative correlation with lymphocyte count (19). Since neutrophils play a dominant role in inflammation, NLR is thought to predict inflammation better than PLR. NLR and PLR are simple and cost effective markers of inflammation when compared with other inflammatory markers such as ILs and TNF-α.

## CONCLUSIONS

As we know, localized testicular tumor has a shorter time interval between cancerogenic effect and formation of a macroscopic tumor, relative to other solid tumors and earlier increase in the number of neutrophils. Low cost, easy accessibility and reproducibility of a whole blood count are the other factors that promote its use in clinical practice. This study indicates the potential usefulness of a new predictor of the disease. The limitations of our study were that it was a retrospective one with limited study group and had not a prognostic predictive design. Larger, randomized controlled studies are needed at this field.
